# Cytomegalovirus Cholangiopathy in an Immunocompetent Patient: A Case Report and Literature Review

**DOI:** 10.1155/2021/2420668

**Published:** 2021-11-10

**Authors:** Ervin Alibegovic, Admir Kurtcehajic, Boris Ilic, Ahmed Hujdurovic, Edinka Smajic, Amar Habibovic, Darja Perkunic

**Affiliations:** ^1^Department of Gastroenterology and Hepatology, University Clinical Center Tuzla, Tuzla 75000, Bosnia and Herzegovina; ^2^Medical Faculty, University of Tuzla, Tuzla 75000, Bosnia and Herzegovina; ^3^Department of Internal Medicine, Medical Center “Plava Poliklinika”, Plava Medical Group, Tuzla 75000, Bosnia and Herzegovina; ^4^Department of Radiology, Medical Center “Plava Poliklinika”, Plava Medical Group, Tuzla 75000, Bosnia and Herzegovina; ^5^Department of Biochemistry, Medical Center “Plava Poliklinika”, Plava Medical Group, Tuzla 75000, Bosnia and Herzegovina

## Abstract

A 37-year-old man presented with jaundice, upper right quadrant pain, and intermittent fever with chills. Laboratory assessment showed biliary stasis, with total bilirubin of 203 *µ*mol/L (2–20), conjugated bilirubin of 105 *µ*mol/L, and alkaline phosphatase of 556 U/L (30–120). Markers for hepatitis A–E viruses were negative. Serology assessment for rubeola, herpes simplex virus, Epstein-Barr virus, and *Toxoplasma gondii* showed negative IgM antibodies. HIV serology status was negative. For cytomegalovirus, both types of antibodies (IgM and IgG) were positive, with an IgM level >300 U/mL. pp65 antigen was also detected as well as CMV DNA. Diagnostic imaging of the abdomen except the dilated common bile duct showed a normal appearance of the gallbladder, liver, pancreas, spleen, and both kidneys. To our knowledge, cytomegalovirus cholangiopathy in the absence of any other underlying disease has not been reported. Therefore, the presence of cholangiopathy in our patient is interesting from an imaging, laboratory, and clinical point of view.

## 1. Introduction

Cytomegalovirus (CMV), also known as human herpesvirus type 5, has a seroprevalence of 30–100%, depending on the age, ethnicity, social, and immune status of the patient. CMV in immunocompetent patients typically manifests as an undifferentiated viral syndrome or a mononucleosis-like illness, which is usually self-limiting. However, in immunocompromised patients, CMV can cause severe complications such as encephalitis, pneumonitis, hepatitis, uveitis, retinitis, colitis, and graft rejection. In immunocompetent patients, the gastrointestinal tract is the primary site of involvement of a CMV infection. Specifically, or in more detail, medical literature has reported colitis and hepatitis in association with CMV infections [1–6].

Acute cholangitis is a morbid condition. It occurs when biliary stenosis, due to various benign causes or the presence of a tumour, results in cholestasis and biliary infection [7, 8]. To our knowledge, CMV cholangiopathy in the absence of any other underlying disease has not been reported. Here, we present the imaging, clinical, and laboratory features of CMV-related acute cholangitis in an immunocompetent patient.

## 2. Case Presentation

A 37-year-old man presented to our hospital with jaundice, upper right quadrant pain, and intermittent fever with chills. The symptoms had arisen 5–7 days before admission. He had no history of severe illness or surgery; his medical history was negative on the medications, alcohol, and tobacco.

On examination, the patient's temperature was 37.8°C, his blood pressure was 130/80 mmHg, his pulse rate was 100 beats per minute, and his respiratory rate was 16 breaths per minute. He was alert and oriented. Nonspecific inflammatory parameters were elevated, patient's white blood cells were 12.7 (4–10 × 10^9^/L), C-reactive protein was 77 (<0.5 mg/dL), and the erythrocyte sedimentation rate was 53 in the first hour. A complete blood count showed the following: red blood cells: 5.1 (4.2–5.4 million/mcL), hemoglobin: 12.5 (12–15 g/dL), hematocrit: 41 (36–47%), and platelets: 380 (150–400 × 109/L).

Laboratory assessment showed biliary stasis, where total bilirubin was 203 *µ*mol/L (2–20), unconjugated bilirubin was 98 *µ*mol/L, and conjugated bilirubin was 105 *µ*mol/L. The results of other liver function tests were as follows: *γ*-glutamyl transferase: 702 U/L (2–30), alkaline phosphatase: 556 U/L (30–120), lactate dehydrogenase: 717 U/L (100–200), aspartate aminotransferase: 258 U/L (10–30), alanine aminotransferase: 207 U/L (10–40), serum amylase: 63 U/L (27–131), international normalized ratio: 1.01 (0.9–1.1), total protein: 6.9 g/dL [6–8], serum albumin: 4.1 g/dL (3.5–5.0), triglycerides: 119 mg/dL (˂160), and cholesterol: 167 mg/dL (˂200). Values for other biochemical tests were within the normal ranges. Markers for hepatitis A–E viruses were negative.

**Figure 1:**
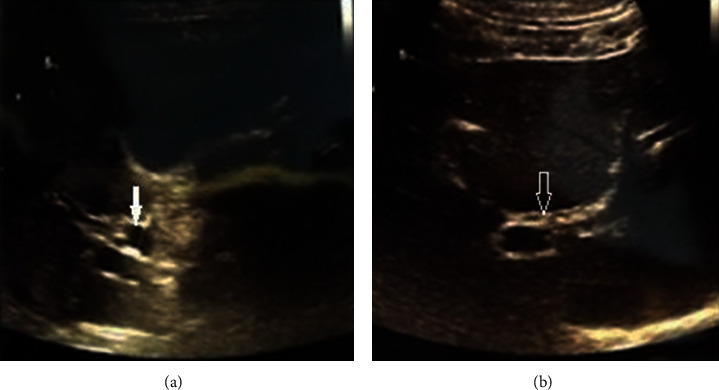
Abdominal ultrasound showing (a) dilated common bile duct (white arrow) (b) on the 12th hospital day normal appearance of common bile duct (arrow).

**Figure 2:**
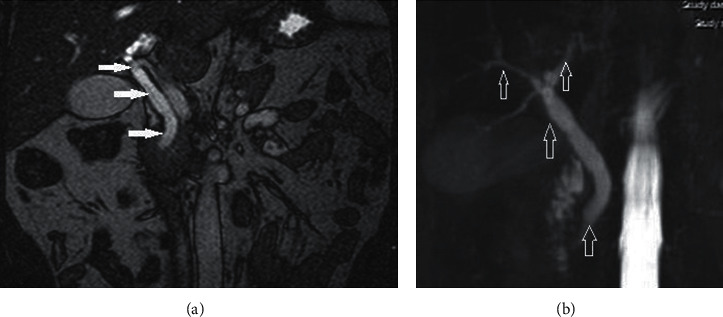
MRI image showing (a) dilation of hepatic and common bile duct (white arrows). MRCP image showing (b) full-length dilation of biliary tree (arrows).

Real-time sonography of the abdomen indicated a dilated common bile duct (CBD; Figure 1(a)), but the gallbladder, liver, pancreas, spleen, and both kidneys appeared normal. Radiology assessment through magnetic resonance imaging (MRI) of the abdomen excluded malignant pathology and lymphadenopathy. Magnetic resonance cholangiopancreatography (MRCP) confirmed whole-length dilatation of the biliary tree and excluded choledocholithiasis (Figure 2). An endoscopy of the upper gastrointestinal tract and duodenal papilla was normal. Colour Doppler echocardiography and a chest X-ray were also normal.

The patient underwent conservative treatment, parenteral infusions, including ciprofloxacin 1 g/day, metamizole 500 mg/day, pantoprazole 40 mg/day, and liver supplements such as silymarin with vitamin B complex. Serology assessment (ELISA assay) for rubeola, the herpes simplex virus, and Epstein-Barr virus showed negative IgM and positive IgG antibodies. A serology assessment for *Toxoplasma gondii* showed negative IgM and IgG antibodies. The patient's HIV serology status was negative. For CMV, both types of antibodies (IgM and IgG) were positive, with an IgM level of 780 U/ml and an IgG level of 350 U/ml.

After 12 days, the patient was asymptomatic. His skin colour returned to normal, and he started eating.

Bilirubin levels normalized, as well as the other liver enzymes. Three weeks after beginning of the symptoms, a follow-up blood sample was again positive for CMV antibodies (IgM 400 and IgG 500). pp65 antigen was also positive, and real-time polymerase chain reaction (PCR) confirmed the presence of CMV DNA. Control sonography of the abdominal organs showed that the CBD had a normal appearance (Figure 1(b)). On day 15, the patient was discharged, with a diagnosis of CMV cholangiopathy. Six months later, a control exam with real-time sonography showed that the biliary tract had a normal appearance, and liver enzymes were also within the normal ranges.

## 3. Discussion

According to the Tokyo guidelines 2018, the diagnostic criteria for acute cholangitis includes systemic inflammation (fever and/or shaking chills) and cholestasis (jaundice, abnormal liver function tests) on diagnostic imaging [7], as imaging findings and laboratory markers are distinct criteria.

On admission, our patient had jaundice, upper right quadrant pain, and intermittent fever, a condition known as Charcot's triad. Biochemical observations indicated hyperbilirubinaemia and increased levels of enzymes associated with biliary stasis.

From the laboratory point of view, interesting feature was more elevated aspartate aminotransferase than alanine aminotransferase, and both bilirubin (indirect/direct) were almost equal; it has not been seen frequently in nonalcoholic and noncirrhotic patients.

Imaging plays a pivotal part in the diagnosis of cholangitis by identifying predisposing factors and revealing complications. Sonography is highly sensitive and specifically used for imaging of the gallbladder and assessing bile duct dilatation [7, 8]. Our diagnostic imaging was based on real-time sonography, MRI, and MRCP, which excluded the presence of stones in the biliary tract, benign strictures, and a possible malignancy as a cause of obstruction. According to the diagnostic criteria in the 2018 Tokyo guidelines, our patient had acute cholangitis, with clear clinical, biochemical, and imaging signs.

The cause of this type of biliary stasis was unclear. Endoscopic retrograde cholangiopancreatography (ERCP) was initially planned as part of the diagnostic and therapeutic protocol, but after a week, the patient felt clinically better, and all bilirubin levels subsequently decreased.

Based on the recommended diagnostic approach, all known causes of acute cholangitis, such as cholelithiasis, benign biliary stricture, malignant occlusion, pancreatitis, duodenal diverticulum, and iatrogenic factors [7, 8], were ruled out. Serological studies indicative of acute CMV infection include the identification of CMV pp65 antigen in blood sample as a marker of active viral replication, positive IgM antibodies, or a significant increase in the titre of IgG antibodies in paired samples obtained during the infection. Active CMV infection is defined by detection of pp65 antigen-positive and/or CMV DNA in the blood [1, 3, 5, 6].

A follow-up blood sample beside the increased level of IgG antibodies and positive pp65 antigen showed the presence of CMV DNA via real-time PCR. After reviewing the patient's medical history and serology status, the only factor that seemed to have a link with this type of cholangiopathy was a CMV infection—pp65 antigenemia and positive IgM and IgG antibodies; CMV DNA was also detected via real-time PCR. Oku and colleagues [9] reported CMV cholangitis in an immunocompetent patient with diabetes mellitus and essential hypertension. Diagnostic imaging (based on computed tomography and ERCP) showed cholelithiasis and a focal narrowing of the distal CBD, followed by pancreatitis. CMV was also detected. The real cause of this type of adverse event remains unclear; it could be the result of cholelithiasis and stricture of CBD or the result of CMV infection. Imaging assessment in our case showed an absence of any strictures of the CBD and no presence of the stone in the complete biliary tract, which could be a predisposing factor for cholangitis.

Jensen and colleagues [4] reported acute CMV hepatitis in a patient with a previous cholecystectomy caused by gallstones. MRCP was performed to address the question of the pathology of the biliary duct system, but this did not show any pathological results. There was no cholangiopathy, and this case did not provide any imaging support.

Regarding the gastrointestinal tract, Pasticci and colleagues [10] reported acute appendicitis due to CMV in an apparently immunocompetent patient. The biliary tract was also involved in this patient; six years previously, the patient had been diagnosed with primary sclerosing cholangitis and ulcerative colitis, with suspected retroperitoneal fibrosis, bile sludge, and splenomegaly. Prasad and colleagues [11] found CMV hemobilia in a patient who had undergone long-term steroid therapy; on admission, the patient presented with hematemesis, fever, and retroperitoneal fluid collection.

Tleyjeh and colleagues [12] reported a case of CMV cholangitis that occurred in a patient undergoing chronic corticosteroid therapy who presented with recurrent hemobilia and biliary obstruction and was successfully treated with ganciclovir and a cholecystostomy.

Unlike these last three cases, aside from CMV cholangiopathy, our patient did not have any other underlying disease and/or the immunosuppressive therapy.

A review article by Rafailidis and colleagues [1] about CMV infection in immunocompetent patients noted possible CMV gastrointestinal complications such as colitis, gastroenteritis, duodenitis, ileitis, proctitis, and hepatitis. However, CMV cholangitis was not present. Therefore, the presence of cholangiopathy in our patient is interesting from an imaging, biochemical, and clinical point of view.

Although it is currently thought that CMV infection in immunocompetent patients does not require treatment, the risks and benefits of specific antiviral treatment for severely ill patients have not been adequately addressed [1]. Our patient had a good response to conservative treatment, which included infusions, antibiotics, liver supplements, and a proton pump inhibitor.

## 4. Conclusion

In conclusion, all patients with onset cholestasis should undergo diagnostic assessment based on a thorough laboratory analysis, different imaging modalities, an upper gastrointestinal endoscopy, and conventional assessment of serology status, checking for hepatitis viruses A–E and HIV. In the absence of clear jaundice aetiology, CMV antibodies, pp65 antigen, and CMV DNA should be considered as part of the diagnostic protocol.

## Data Availability

The data used to support the findings of this study are included within the article.

## Abbreviations

CBD: Common bile duct
CMV: Cytomegalovirus
ERCP: Endoscopic retrograde cholangiopancreatography
HIV: Human immunodeficiency virus
MRCP: Magnetic resonance cholangiopancreatography
MRI: Magnetic resonance imaging.
